# General Anesthesia as a Multimodal Individualized Clinical Concept

**DOI:** 10.3390/medicina58070956

**Published:** 2022-07-19

**Authors:** Alexandru Florin Rogobete, Dorel Sandesc

**Affiliations:** 1 Department of Anaesthesia and Intensive Care, “Victor Babes” University of Medicine and Pharmacy, 300041 Timisoara, Romania; dsandesc@yahoo.com; 2 Clinic of Anaesthesia and Intensive Care, Emergency County Hospital “Pius Brinzeu”, 325100 Timisoara, Romania

**Keywords:** entropy, bispectral index, multimodal monitoring, general anesthesia, electroencefalography

## Abstract

In the last decades, several new and modern techniques have been developed for the continuous monitoring of vitals for patients undergoing surgery under general anesthesia. These complex methods are meant to come as an adjunct to classical monitoring protocols used in general anesthesia to increase patient safety. The main objectives of multimodal monitoring are avoiding the over- or underdosing of anesthetic drugs, adapting the concentration for the substances in use, reducing post-anesthetic complications, and increasing patient comfort. Recent studies have shown a series of benefits with significant clinical impact such as a reduced incidence of nausea and vomiting, shorter reversal times, a reduction in opioid consumption, shorter hospital stays, and an increase in patient satisfaction.

This Special Issue, “General Anesthesia as a Multimodal Individualized Clinical Concept”, in the *Medicina* journal of MDPI’s “Intensive Care/Anesthesiology” section, reports international studies regarding the concept of the personalized monitoring of patients under general anesthesia. Furthermore, it describes modern monitoring techniques for certain anesthesia-specific parameters such as the degree of hypnosis, continuous monitoring of the nociception–antinociception balance, neuromuscular transmission monitoring, and hemodynamic monitoring (heart rate, invasive or non-invasive measurement of blood pressure, peripheral oxygen saturation, temperature). This Special Issue also describes new techniques for monitoring respiratory gases perioperatively by using modern technology such as indirect calorimetry.

Cotae et al., in a randomized prospective study have analyzed the impact that monitoring the degree of hypnosis by using the Entropy technology (E-Entropy Module, GE Healthcare, Helsinki, Finland) and the nociception–antinociception balance through Surgical Pleth Index (SPI Module, GE Healthcare, Helsinki, Finland) can have on postoperative delirium and cognitive dysfunction (POCD) in 107 trauma patients. For the statistical analysis, the authors used two study groups. The first was the target group, in which general anesthesia management was based on multimodal monitoring, and the second group that received classical monitoring in accordance with international guidelines. In the multimodal monitoring group, they studied both Entropy and SPI as constants throughout the general anesthesia. Patient assessment for POCD was based on the Neelon and Champagne (NEECHAM) Confusion Scale. Following this study, they identified statistically significant differences (*p* < 0.05) between the two groups regarding the incidence of POCD, although in the intervention group the overall number was significantly lower [1].

In more detail, Rogobete et al., in their review article "*Multiparametric Monitoring of Hypnosis and Nociception–Antinociception Balance during General Anesthesia—A New Era in Patient Safety Standards and Healthcare Management*", described a series of modern techniques currently in use in the clinical setting for personalized and individualized general anesthesia monitoring. The group described and summarized information on the most modern monitoring techniques for the degree of hypnosis (Bispectral Index, BIS, Medtronic-Covidien, Dublin, Ireland; Response Entropy/State Entropy, Entropy, GE Healthcare, Hesinki, Findland; Narcotrend index, NCT, Monitor Technik, Germany; and composite auditory evoked potential index, cAAI, AEP Monitor/2, Danmeter A/S, Odense, Denmark). They have also presented recent studies on the topic and have shown the impact of these techniques on hemodynamic stability, incidence of adverse events, anesthetic drug consumption and other quality and safety indicators in medical practice. Furthermore, this review article describes different monitoring techniques for the nociception–antinociception balance and for neuromuscular transmission. The authors also bring to light the impact of general anesthesia on the systemic inflammatory status, oxidative stress, and other biochemical pathways directly or indirectly involved in the clinical outcome of patients undergoing surgery under general anesthesia [2].

Tiglis et al. have published an article—"*Incidence of Iron Deficiency and the Role of Intravenous Iron Use in Perioperative Periods*"—that shows the importance of the multidisciplinary monitoring of patients undergoing general anesthesia. The research group describes a series of mechanisms and biochemical pathways associated with iron deficit and preoperative anemia, as well as with post-operative low iron levels. Their article presents the impact on clinical prognosis, the direct association between iron deficiency and perioperative need for blood transfusion, incidence of postoperative infection, ICU length of stay, morbidity and mortality, and the economic impact of the medical act [3].

Balan et al., in a review article on ultrasound-based monitoring and diagnosis techniques, underline the importance of ultrasound-guided regional anesthetic techniques on the management of nociception–antinociception balance and the impact of these techniques on opioid consumption, patient satisfaction, and postoperative recovery [4].

Fiedler et al., in an original article, have analyzed a method that is frequently used in ventilatory support for patients under general anesthesia. They carried out an observational trial aiming at evaluating the impact of positive end-expiratory pressure (PEEP) levels on ventilation parameters and gastric air insufflation during laryngeal mask general anesthesia in children. The study only included pediatric patients (n = 67), aged 1 to 11. The authors identified statistically significant differences for ventilatory parameters such as: peak pressure (*p* < 0.05), tidal volume (*p* < 0.05), and dynamic compliance (*p* < 0.05). They reported an increase in all parameters that are directly influenced by the increase of PEEP, except from etCO_2_, for which they reported a significant increase, and for respiratory rate, for which no differences have been reported. They have also identified a proportional increase in gastric insufflation with increased PEEP. The authors have therefore proven the importance of multimodally monitoring mechanical ventilation during general anesthesia, as well as the fact that modern techniques can reduce side-effects associated with anesthesia [5].

An interesting article, adapted to the crisis that was generated by the COVID-19 pandemic, has been published by Secosan et al., who report on the impact of disinformation regarding SARS-CoV-2 and the impact the pandemic had on the medical personnel. The authors included in their study 100 employees of the Clinic for Anesthesia and Intensive Care in “Pius Brinzeu” Emergency County Hospital in Timisoara, Romania. They all received a questionnaire between March and April 2020 that was meant to evaluate the degree of depression, anxiety, stress, and the incidence of insomnia. The study identified the negative impact that social disinformation had on the stress and anxiety levels of the medical personnel, overlapping with overtime during the crisis, the great number of patients, social and medical drama, the very high number of deaths, and being mentally and physically overworked [6].

In conclusion, this Special Issue presents a number of modern monitoring techniques for all segments of general anesthesia and current clinical practice, presenting updates in the field of monitoring of degree of hypnosis, perioperative pain, neuromuscular transmission, hemodynamic stability, ventilatory support, and the most important biochemical pathways associated with inflammation. Moreover, adapted to the ongoing COVID-19 pandemic, the time of the Special Issue’s publication has proven the importance of periodic evaluation of the psychological well-being of medical personnel, as well as the importance of offering psychological support.

## Data Availability

Not applicable.

## References

[1] Cotae A.-M., Ţigliş M., Cobilinschi C., Băetu A., Iacob D., Grinţescu I. (2021). The Impact of Monitoring Depth of Anesthesia and Nociception on Postoperative Cognitive Function in Adult Multiple Trauma Patients. Medicina.

[2] Rogobete A., Bedreag O., Papurica M., Popovici S., Bratu L., Rata A., Barsac C., Maghiar A., Garofil D., Negrea M. (2021). Multiparametric Monitoring of Hypnosis and Nociception-Antinociception Balance during General Anesthesia—A New Era in Patient Safety Standards and Healthcare Management. Medicina.

[3] Țigliș M., Neagu T., Niculae A., Lascăr I., Grințescu I. (2020). Incidence of Iron Deficiency and the Role of Intravenous Iron Use in Perioperative Periods. Medicina.

[4] Balan C., Bubenek-Turconi S.-I., Tomescu D., Valeanu L. (2021). Ultrasound-Guided Regional Anesthesia–Current Strategies for Enhanced Recovery after Cardiac Surgery. Medicina.

[5] Fiedler M., Schätzle E., Contzen M., Gernoth C., Weiß C., Walter T., Viergutz T., Kalenka A. (2020). Evaluation of Different Positive End-Expiratory Pressures Using Supreme™ Airway Laryngeal Mask during Minor Surgical Procedures in Children. Medicina.

[6] Secosan I., Virga D., Crainiceanu Z., Bratu L., Bratu T. (2020). Infodemia: Another Enemy for Romanian Frontline Healthcare Workers to Fight during the COVID-19 Outbreak. Medicina.

